# Recent Selection on a Class I *ADH* Locus Distinguishes Southwest Asian Populations Including Ashkenazi Jews

**DOI:** 10.3390/genes9090452

**Published:** 2018-09-07

**Authors:** Sheng Gu, Hui Li, Andrew J. Pakstis, William C. Speed, David Gurwitz, Judith R. Kidd, Kenneth K. Kidd

**Affiliations:** 1 Department of Genetics, School of Medicine, Yale University, New Haven, CT 06520, USA; sheng_gu@yahoo.com (S.G.); andrew.pakstis@yale.edu (A.J.P.); william.speed@yale.edu (W.C.S.); judith.kidd@yale.edu (J.R.K.); 2 Ministry of Education Key Laboratory of Contemporary Anthropology, School of Life Sciences, Fudan University, Shanghai 200433, China; lhca@fudan.edu.cn; 3 Department of Human Molecular Genetics and Biochemistry, Faculty of Medicine, Tel Aviv University, Tel Aviv 69978, Israel; gurwitz@post.tau.ac.il

**Keywords:** alcohol dehydrogenase, population genetics, genetic selection

## Abstract

The derived human alcohol dehydrogenase (ADH)1B*48His allele of the *ADH1B Arg48His* polymorphism (rs1229984) has been identified as one component of an East Asian specific core haplotype that underwent recent positive selection. Our study has been extended to Southwest Asia and additional markers in East Asia. *F_st_* values (Sewall Wright’s fixation index) and long-range haplotype analyses identify a strong signature of selection not only in East Asian but also in Southwest Asian populations. However, except for the ADH2B*48His allele, different core haplotypes occur in Southwest Asia compared to East Asia and the extended haplotypes also differ. Thus, the ADH1B*48His allele, as part of a core haplotype of 10 kb, has undergone recent rapid increases in frequency independently in the two regions after divergence of the respective populations. Emergence of agriculture may be the common factor underlying the evident selection.

## 1. Introduction

The human alcohol dehydrogenase (*ADH*) gene cluster has been widely studied for association with diseases, especially alcoholism [1,2,3] and for population diversity studies [4,5,6,7,8]. The protective effect against alcoholism of the ADH1B*48His (previously named ADH2*2) allele at rs1229984 is considered one of the most strongly confirmed associations [1,9,10,11,12]. Also strongly confirmed is the evidence that the derived-protective allele (ADH1B*48His) has undergone recent positive selection in East Asia [13,14,15,16,17]. Different geographic regions differ in the frequencies of the genetic polymorphisms in *ADH1B* and *ADH1C*, the genes for the primary ethanol metabolizing enzymes [18,19]. We originally found that the ADH1B*48His allele reaches high frequencies not only in East Asia but also in Southwest Asia, while the frequency of this derived allele remains lower between these two geographic regions [20,21]. We also found ethnic-specific variation in the haplotypes with ADH1B*48His within East Asia and different haplotypes in Southwest Asia [14]. Subsequently, Peng et al. [15] has associated the rise of the allele frequency with domestication and spread of rice. We speculated that this derived allele increased in frequency independently in East Asia and Southwest Asia after humans had spread across Eurasia. We suspected that the *ADH1B* locus in Southwest Asia had also undergone positive selection. Therefore, we undertook to examine this region using the long-range haplotype (LRH) test for populations in Southwest Asia [22].

**Figure 1:**
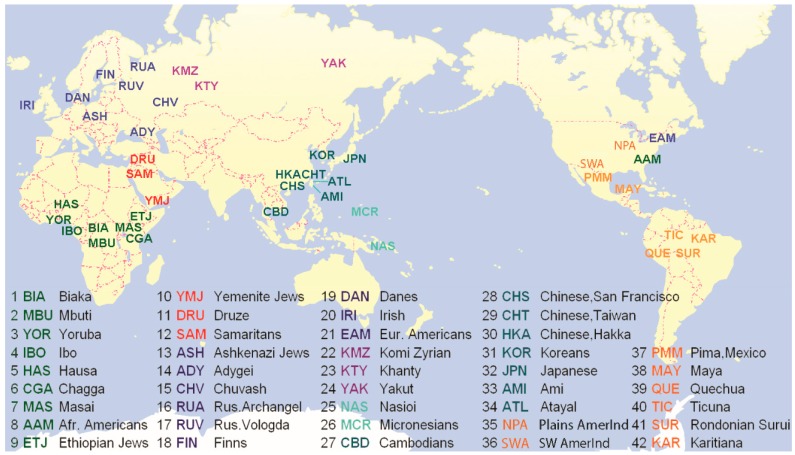
All 42 populations used in this study. Populations are geographically categorized and colored into eight groups: Africa (BIA, MBU, YOR, IBO, HAS, CGA, MAS, AAM, and ETJ), Southwest Asia (YMJ, DRU, and SAM), Europe (ASH, ADY, CHV, RUA, RUV, FIN, DAN, IRI, and EAM), Siberia (KMZ, KTY, and YAK), Pacific Islands (NAS and MCR), East Asia (CBD, CHS, CHT, HKA, KOR, JPN, AMI, and ATL), North America (NPA, SWA, PMM, and MAY,) and South America (QUE, TIC, SUR, and KAR). The three-character population symbols and corresponding full names are listed in pairs.

**Figure 2:**
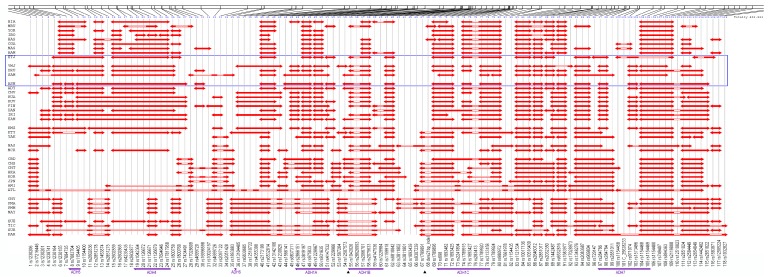
The schematic representation of regions of high linkage disequilibrium (LD) across the human alcohol dehydrogenase (*ADH*) clusters. HAPLOT and the default *r*^2^ algorithm were used. All 118 genetic markers (including one insert-deletion site treated as a single nucleotide polymorphism (SNP)) are listed at the bottom. The locations of two short tandem repeat polymorphisms (STRP) loci are indicated at the bottom by the black triangles. The arrow above each gene symbol indicates the downstream direction; the region is plotted from centromere to telomere. Generally speaking, populations of the same group have similar LD structure with some variation. Highlighted within the blue box, Southwest Asian populations show LD patterns similar to those of European and African populations.

Initially, we studied a global sampling of 42 populations (Figure 1, additional information in Table S1) and examined the linkage disequilibrium (LD) pattern across the whole *ADH* gene cluster in all populations (Figure 2). A total of 118 single nucleotide polymorphisms (SNPs) were genotyped in this genomic region (Figure 2). For the detection of selection, we focus on Southwest Asian populations since selection in East Asian populations has been well documented. Three southwest Asian populations, Yemenite Jews (YMJ), Druze (DRU), and Samaritans (SAM), were initially selected. Considering the genetic proximity of Ethiopian Jews (ETJ) to those Southwest Asian populations, as well as the original geographic origins of Ashkenazi Jews (ASH) [23,24,25,26], we also extended our study to these two populations. Though geographically ETJ belong to a different continent and ASH have lived in Europe for some time, we shall refer to all five populations, YMJ, DRU, SAM, ETJ, and ASH, collectively as Southwest Asia.

No single algorithm is able to capture all possible signatures of positive selection. We applied both *F_st_*(Sewall Wright’s fixation index) [27] and LRH [22] analyses to our data. An unusually high *F_st_* can be the signature of local positive selection driving substantial changes in allele frequencies [28,29], and the LRH analysis, including the extended haplotype homozygosity (EHH) test and Relative EHH (REHH) test, detects a rapid rise in haplotype frequency interpreted as detecting an allele under positive selection that has recently been rapidly driven to high frequency and tends to lie on an extended haplotype with low diversity [22]. However, high *F_st_* values can occur in the absence of selection [30] and positive LRH analysis is considered to be a stronger indication of selection.

## 2. Materials and Methods

### 2.1. Subjects

Over 2100 individuals from a global sample of 42 populations have been typed for this study for a total of 118 SNPs. These samples have been described previously [13] and descriptions can be found in ALFRED [31,32] through links for any of the polymorphisms in this study. These populations were categorized into eight geographic groups (Figure 1 and Table S1), with Southwest Asia of particular interest. Three populations, YMJ, DRU, and SAM, are geographically categorized as Southwest Asian populations, while ETJ and ASH, though geographically grouped into Africa and Europe respectively, were analyzed together with YMJ, DRU, and SAM because of genetic similarity and high frequency of the target allele, ADH1B*48His. Subsequent to the original findings arguing for selection in Southwest Asia, two additional sets of populations were obtained and studied. The confirmatory samples of Ethiopian Jews (ETJ2), Ashkenazi (ASH2), and Palestinian Arabs (PAL) (Table S1) were obtained from the National Laboratory for the Genetics of Israeli Populations and studied for 10 markers flanking the core. Additional populations have been collected and typed for the two focal SNPs, rs1229984 and rs3811801, to obtain a wider picture of the allele frequency variation of the derived alleles.

### 2.2. Polymorphic Sites around ADH Clusters

*ADH* cluster genes in our study include *ADH7*, *ADH1C*, *ADH1B*, *ADH1A*, *ADH4*, and *ADH5*. A total of 118 SNPs were studied, which extend across ~453 kb with a density of ~1 SNP per 3.8 kb (Figure 2). This more than doubles the number of markers that were included in the earlier selection studies in East Asian populations [13,14]. Most polymorphic sites were genotyped by TaqMan® (AppliedBiosystems, ThermoFisher Scientific, Waltham, MA, USA) while the rest were typed by custom chip from Illumina Inc (San Diego, CA, USA), fluorescence polarization, and PCR-based RFLP (restriction fragment length polymorphism) methods. For each marker, the typing was complete for at least 90% of the individuals in all populations. Allele frequencies were estimated by gene counting and all frequencies can be found in ALFRED [31,32]. For each site, the average heterozygosity was calculated and the Hardy–Weinberg (HW) test was applied. Out of all 118 markers, there are three sites whose average heterozygosity falls below 0.05. However, in some populations, the heterozygosity of these markers is as high as 0.40. Sporadically ~1.9% of all HW tests, considering all SNPs in all populations, failed at a significance level of 5% and ~0.4% of HW tests failed at a significance level of 1%. Therefore, these failures of HW test are well below expectation, and we consider our data sufficiently sound for the studies conducted.

### 2.3. Linkage Disequilibrium Pattern

We examined the LD pattern mainly through the haplotype block structure using the program HAPLOT [33]. The Kidd *r*^2^ definition [33] has been used for block partition. Previous studies [34] have shown that Kidd *r*^2^ block partition algorithms best preserve the consistency of within-group population haplotype block structure. Here, we are referring to a haplotype block purely as a region of high LD instead of a fundamental aspect of the genome. The same genomic coverage of blocks in different populations may be composed of different alleles.

### 2.4. Haplotype Inference

Long range haplotype inference from our phase-unknown genotyping data was achieved through fastPHASE [35], which is a Markov Chain haplotyper free of convergence limitation associated with traditional expectation maximization (EM) algorithm-based haplotypers. Sequences of haplotypes were then loaded for test of recent positive selection. However, empirical studies suggest HAPLO [36], an EM algorithm based haplotyper, shows superiority in capturing rare haplotypes especially in the case of missing data. Thus, for our core haplotype (only a few markers) inference, we applied HAPLO for core haplotype pattern summary. The results of the core haplotype frequencies by using these two different haplotypers turn out to be very close.

### 2.5. Test for Recent Positive Selection

We calculated *F_st_* [27] values for all 118 sites, and high *F_st_* value is an indication of, though not necessarily the signature of, positive selection. Then we applied LRH test [22] to haplotype sequences in each population with preselected core haplotypes. EHH and REHH values over large distances would be strong evidence of recent selection. A large collection of simulated haplotypes, which assume neutral evolution with no selection, were used for REHH calculations as reference points of no selection. The REHH values from our real population core haplotypes were then plotted against those from simulated haplotypes. Since the data are not normally distributed, we were unable to apply parametric statistical tests. Instead, we separated all data points into 20 bins (5% interval per bin) and then calculated the 50th, 75th, and 95th percentile curves for delimitation. An REHH value above 95th percentile would be considered a positive result for selection.

### 2.6. Inference on Human Evolution

Since the selection on the *ADH1B* locus is presumably independent in Southwest Asia and East Asia, we are interested in estimating the approximate time when the mutant allele as well as its associated haplotype first arose, and in estimating the strength of selection on the selected haplotype following the age estimation. We selected several SNPs in this 26 kb interval (Arg48His (rs1229984)—rs1159918—rs6810842—rs3811802—rs3811801—rs1693439—rs9307239) for analysis. These define haplotypes that previous results showed were either under selection in East Asia or were at elevated frequency in Southwest Asia [14,20,21]. In addition to the SNPs we typed two short tandem repeat polymorphisms (STRPs). Short tandem repeat polymorphisms are short, identical sequences of DNA (two, three, four, or more nucleotides in length) that are tandemly repeated a variable number of times. We studied a (GTAT)n 13,475 bp upstream of the Arg48His site and a (TA)n 12,940 bp downstream of that site (Figure 2 and Table 1) on all individuals. Repeat numbers are based on product sizes for the primers used in comparison with publicly available sequences. Haplotypes were estimated using PHASE [37].

### 2.7. Simulations

Haplotypes were simulated using the ms program of Hudson [38] to provide an estimate of what REHH values might be expected for comparison with the observed REHH values. Three previously published demographic scenarios [13], all of which assume neutral evolution without selection, were employed to generate our reference points. The scenarios differ primarily in their population expansion modes: a constant effective population size of 10,000 and two scenarios with a bottleneck event of 2000 starting 2500 generations ago followed by an expansion starting 500 generations ago. In one of the bottleneck expansion scenarios the expansion was instantaneous to 100,000 and in the other it was an exponential population growth to that maximum. Multiple core haplotype frequencies were simulated and the resulting reference points were categorized into 20 bins at core haplotype frequency intervals of 0.05, and three reference curves were plotted using the 50th, 75th, and 95th percentiles.

## 3. Results

### 3.1. Linkage Disequilibrium Pattern

Linkage disequilibrium patterns tend to be similar within each geographic region. African, Southwest Asian, and European populations share a similar overall pattern of regions of high LD (Figure 2). However, the similar regions of high LD in different geographic regions may not necessarily have similar underlying haplotypes [39,40]. The haplotype composition and frequency distribution could be significantly different across geographic locations. Therefore, the similarity of high LD regions among Southwest Asian, African, and European populations does not provide conclusive evidence of genetic similarity among populations in these geographic regions. The haplotype patterns and frequency differences across the geographic regions presented later make this clear.

### 3.2. Fixation Index Distribution

**Figure 3:**
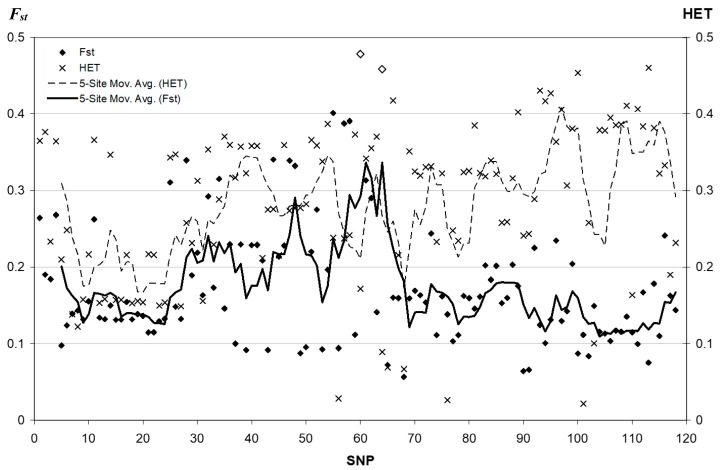
The heterozygosity (HET) and *F_st_* (Sewall Wright’s fixation index) of each genetic marker. Most markers have moderate heterozygosity with only three below 0.05. The 5-site moving average curve of heterozygosity suggests a level of 0.2 to 0.4. The two highest *F_st_* values marked by the empty diamond symbols are *ADH1B* Arg48His/rs1229984 and rs3811801. The 5-site moving average curve of *F_st_* values, drawn as a solid line, peaks around those two SNPs. The *F_st_* of *ADH1B* Arg48His is 3.61 SDs (standard deviation) above the mean, while that of rs3811801 is 3.38 SDs above the mean. Three nearby SNPs, #55 = rs1042026 (*F_st_* = 0.401), #57 = rs2066701 (the *Rsa*I restriction site) (*F_st_* = 0.388), and #58 = rs2075633 (*F_st_*= 0.391), have *F_st_* values > 2.5 SD above the mean.

With the addition of more SNPs to our previous analyses [13,14] we have plotted the *F_st_* values for all 42 populations. The new data did not add any new SNPs with *F_st_* values as high as the five previously identified (Figure 3). SNP #60 (rs1229984/Arg48His, *F_st_*_=_ 0.478) and SNP #64 (rs3811801, *F_st_*_=_ 0.458), marked by the empty diamond symbols, have the highest *F_st_* values. 

Because the frequency of the derived ADH1B*48His allele was already known to have a high-frequency in East Asia, we repeated the *F_st_* calculations omitting the eight East Asian populations and compared them to the distribution of 2554 SNPs on the same individuals in the same 34 populations. Though higher values occur elsewhere in the genome, within the *ADH* cluster the Arg48His/rs1229984 SNP continued to have the highest *F_st_* (0.283) and this value is in 94th percentile for the set of 2554 SNPs. This finding suggests that the Arg48His SNP is still highly differentiated in the remaining 34 populations, and the source of, as well as the reason for this high frequency is of our special interest. This localized genomic region thus shows a potential signature of recent selection. We based our selection of a core region for the LRH analyses upon these *F_st_* findings.

### 3.3. Core Haplotype Pattern

**Figure 4:**
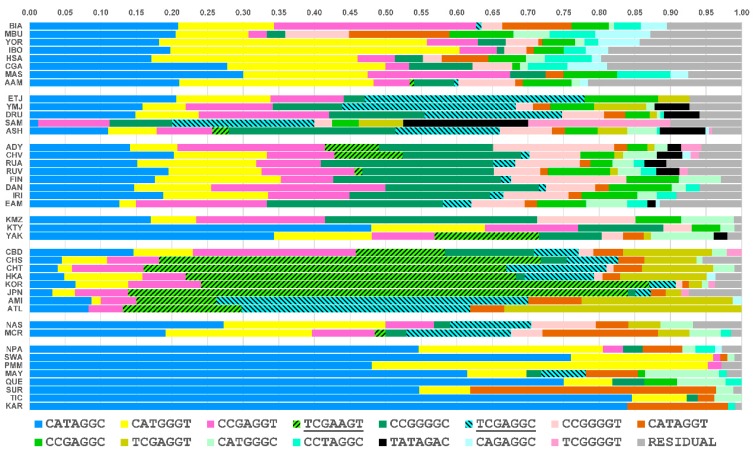
The core haplotype pattern based on seven SNPs (see Table 1) is shown for 42 populations. Haplotype frequencies are highly variable across all geographic regions with Africans displaying the most variation while Native Americans have the least variation. The haplotype TCGAGGC is observed at very common frequencies in Southwest Asians, Ashkenazi, and Ethiopians. In East Asia and the Pacific region this haplotype has comparable frequencies. Another haplotype, TCGAAGT, is predominant in East Asian populations with frequency estimates ranging from 45 to 70% in Han, Koreans, and Japanese. The haplotype TCGA (from TCGAAGT) has shown a signature of recent positive selection in East Asian populations in earlier studies [13,14,15].

We selected SNPs #60-66 in Figure 1 (rs1229984, rs1159918, rs6810842, rs3811802, rs3811801, rs1693439, and rs9307239) as our core to estimate haplotypes for the LRH test (Table 1). This region shows no evidence of recombination and includes both of the high *F_st_* sites, rs1229984 and rs3811801. The haplotype frequencies for this seven SNP core are shown in Figure 4 for all 42 populations. A threshold of 10% was used to group uncommon haplotypes into a residual class. Of all haplotypes, the haplotypes TCGAAGT and TCGAGGC (here the underscored nucleotides correspond to rs1229984 and rs3811801, respectively) are of special interest. Both haplotypes contain the protective ADH1B*48His allele (T), which is in high frequency only in Southwest Asia and East Asia. The haplotype TCGAAGT (green bar with forward slash) is East Asian-specific; our previous studies showing positive selection in East Asian populations used at least three of these SNPs, including the two underlined [13,14]. The haplotype TCGAGGC (light blue bar with backward slash) is common in Southwest Asia and accounts for the high frequency of the ADH1B*48His allele at rs1229984 in that region. We have previously shown that the haplotype containing this allele at rs1229984 has an ethnic-specific distribution in East Asia [14]. The distribution of the haplotype TCGAGGC is not uniform along the pathways of human expansion out of Africa. In sub-Saharan Africa and Europe, the haplotype TCGAGGC is not seen or rare. However, it occurs frequently in Southwest Asia and at comparable frequencies in East Asia and in the Pacific Islands. The specific haplotypes and their frequencies reveal appreciable differences among Southwest Asian, African, and European populations that were not evident from the shared regions of high LD (Figure 2).

### 3.4. Haplotype Homozygosity and Relative Haplotype Homozygosity

**Figure 5:**
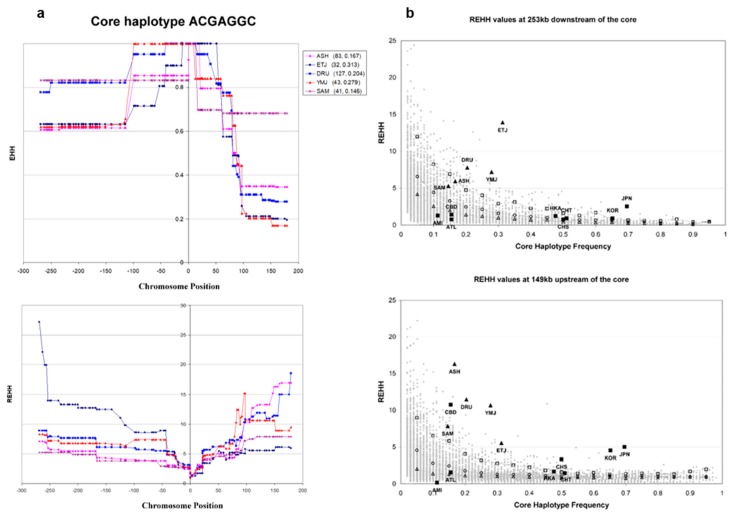
Extended haplotype homozygosity (EHH) and Relative EHH (REHH) plots in Southwest Asian and East Asian populations. The core haplotype position is defined to be zero. The left side of the core is toward downstream direction, while the right side of the core is toward upstream direction. (**a**) EHH and REHH results for the core haplotype TCGAGGC in Southwest Asian populations. Population abbreviations as well as corresponding sample size and the frequency of the selected core haplotype are listed in the middle. All five populations show that, in the downstream direction, the EHH extends over 250 kb from the core haplotype at a level above 0.6. In the upstream direction, however, the EHH only extends around 80 kb at a level above 0.6. The REHH plot reveals a V shape, which means REHH increases continuously from the core to either direction. This is a typical sign of potential recent selection on the target core. (**b**) REHH values from Southwest Asia and E Asia against those from simulated haplotypes. On the left are the REHH values sampled from 253 kb downstream of the core against simulated reference points. Clearly, the REHH of ETJ, DRU, and YMJ is well above the 95th percentile, and that of ASH rides on the 95th percentile curve. Only SAM falls between 75th and 95th percentile. However, the REHH values of East Asian populations are mostly below 95th percentile except JPN (above the line) and KOR (on the line). On the right are the REHH values sampled from 149 kb upstream of the core against simulated reference points. All Southwest Asian populations have REHH above 95th percentile. In East Asia, JPN, KOR, CHS, and CBD have REHH above 95th percentile, while HKA and CHT have REHH on the 95th percentile line. However, the REHH of AMI and ATL fall below 75th percentile.

The EHH and REHH curves of the core haplotypes TCGAGGC in Southwest Asia are plotted in Figure 5. In Southwest Asia, the core TCGAGGC extends 250 kb downstream to a minimum EHH of 0.6, and upstream to around 80 kb at an EHH of at least 0.6 (Figure 5a). In either direction from the core, the REHH of all five populations gradually increases over distance to at least 150 kb telomeric, and at both ends the REHH reaches a minimum of five.

In East Asia the previously identified core haplotype continues to show evidence of selection [13,14]. We noticed a systematic decrease of REHH approximately 150 kb telomeric of the core in East Asian populations in the direction of *ADH7*, suggesting a recombination hot-spot near that location. Therefore, for comparison between Southwest Asian and East Asian core haplotypes against simulated reference points we picked the REHH value of a polymorphic site right before the location at which the quick drop of REHH in East Asia occurs.

The REHH values sampled at 253 kb downstream of the core haplotypes suggest that all Southwest Asian populations except SAM have REHH at or above the 95th percentile. The REHH values sampled at 149 kb telomeric of the core haplotypes suggest that all Southwest Asian populations have REHH above the 95th percentile. Compared with East Asian populations that show only one-sided REHH increase, Southwest Asian populations show a signature of selection on both sides of the core [13,14,15].

### 3.5. Independent Selection

**Figure 6:**
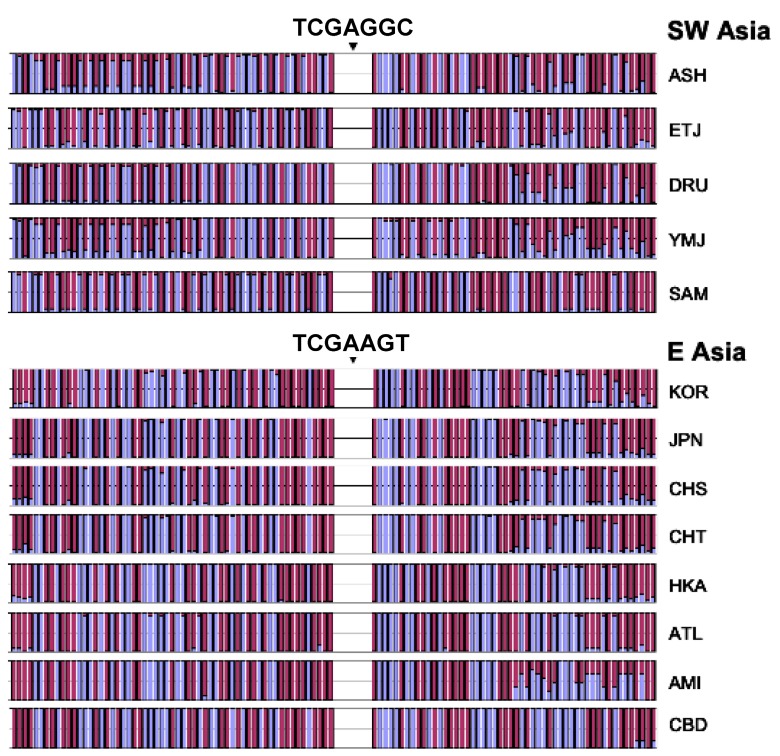
The allele profiles of SNPs in the flanking regions of the core haplotype under selection. Two different colors in each bar represent the relative percentage of two different alleles. Populations of the same geographic location tend to share similar allele profiles, while populations in two different areas apparently differ in allele profiles except occasional similarity.

**Figure 7:**
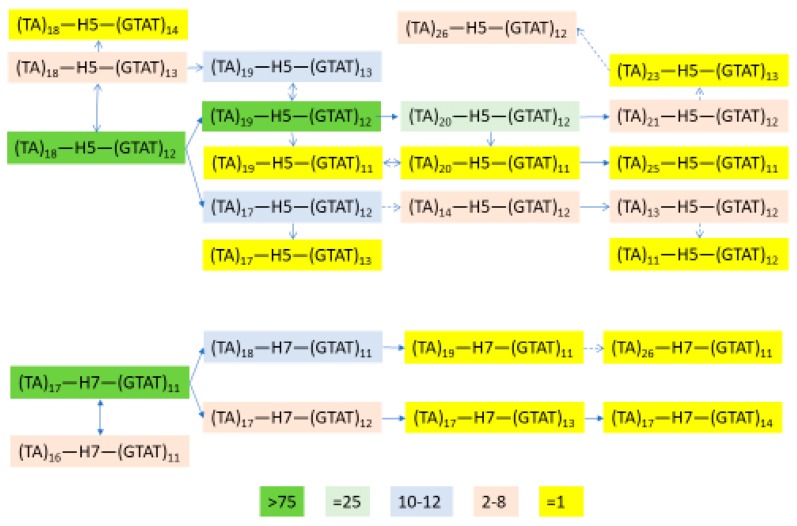
Schematic of STRP evolution flanking the two haplotypes with evidence of selection. The 25 STRP haplotypes for H5 and H7 in the populations studied are shown; color highlighting indicates their frequency counts. See Table S2 for more details. H5 = TCGAGGC in Figure 4; H7 = TCGAAGT in Figure 4. The core haplotypes correspond to H5 and H7 in Li et al. [41]; the shorter core in this study does not allow H6 to be distinguished. Given the assumed mutation rates at the STRPs, these are consistent with recent selection on the chromosomes and compatible with selection associated with the origins of agriculture.

One further step is to examine whether the selection has operated in the two geographic groups independently. The selection could have occurred before or after the divergence of East Asian and Southwest Asian populations. If the selection occurred before the divergence, we would expect to see that the two core haplotypes, TCGAGGC (H5) in Southwest Asia and TCGAAGT (H7) in East Asia, would have similar extended haplotypes, i.e., the alleles at the flanking sites would tend to be the same except for subsequent mutations and an approach to randomness with distance from the core because of subsequent recombination. However, if the selection events are independent and subsequent to the divergence, the extended haplotypes would have much less similarity between regions but be more homogeneous within each region. Our observation suggests that the overall pattern of allele components is different in these two geographic groups despite occasional similarity at some SNPs (Figure 6). The flanking STRPs are also different and their evolution is independent on the two core haplotypes (H5 and H7) (Figure 7). Therefore, we conclude that the selection has occurred independently in East Asia and Southwest Asia after the populations diverged.

### 3.6. Independent Mutation

Given the near absence of the ADH1B*48His allele in the South-Central parts of Asia and the lower frequencies in Central Asia, the possibility of separate mutations arising in Southwest and East Asia needs to be considered. This seems unlikely based on the low prior probability of recurrent mutation. In addition we note that the alleles for the SNPs between rs1229984 and rs3811801 are the same in haplotype H5 and H7, as is the allele at rs1693439. The previous paper by Li et al. [20] involved additional flanking SNPs and similarly concludes that a single mutation event was responsible for the 48His allele globally. We conclude that the ADH1B*48His allele arose once in the population ancestral to populations in both regions. It likely remained at low frequency for some time during which recombination and mutation occurred, altering alleles at nearby sites on 48His-encoding chromosomes. When the selective pressures arose, the rare 48His-encoding chromosomes became common in the separate regions elevating the diverged flanking regions.

### 3.7. Confirmation of Haplotype Homozygosity and Relative Haplotype Homozygosity Patterns in Independent Samples

**Figure 8:**
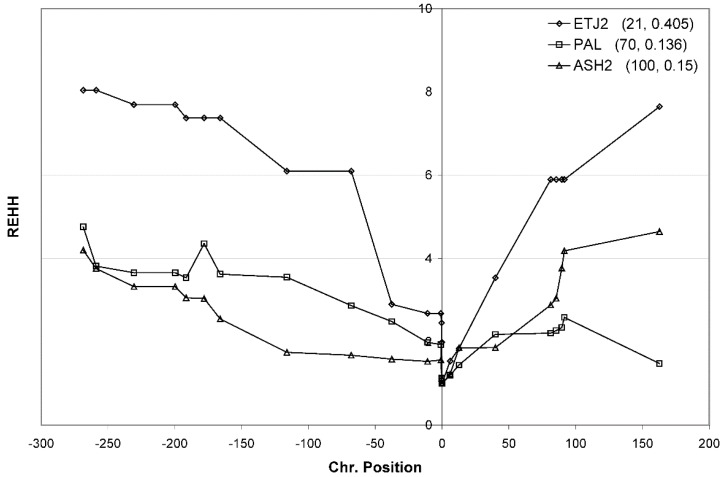
The REHH of three new confirmatory populations, Ethiopian Jews (ETJ2), Ashkenazi (ASH2), and Palestinian Arabs (PAL). At lower SNP density but approximately similar distance from the core, all populations show REHH increases over distance.

Though the signature of selection was clear in four different population samples, we wanted to have different Southwest Asian populations and samples tested to confirm our conclusion on selection on the *ADH1B* locus in Southwest Asians. Therefore, we obtained three new samples of Southwest Asians: a different sample of Ethiopian Jews (ETJ2, *N* = 21), a different sample of Ashkenazi Jews (ASH2, *N* = 100), and a sample of Palestinians (PAL, *N* = 70). We randomly selected 10 markers on either side of the core to genotype in these samples along with the core markers. With a much lower SNP density, we are still able to see a V shape in the REHH curves in all new population samples (Figure 8). The results of ETJ2 and ASH2 are consistent with ETJ and ASH, and PAL gives a pattern similar to other Southwest Asian populations. Thus, we obtained consistent results and can confidently conclude that Southwest Asian populations show a signature of recent selection on the *ADH1B* locus. 

## 4. Discussion

Among a total of 118 SNPs and 42 populations in the main study, *F_st_* values for only two SNPs are >3 standard deviation (SD) above the mean of *F_st_* values: *ADH1B Arg48His*/rs1229984 (0.478) and rs3811801 (0.458). Three other SNPs that are immediately centromeric of rs1229984, rs2075633 (*F_st_* = 0.391), rs2066701 (the *Rsa*I restriction site) (*F_st_* = 0.388), and rs1042026 (*F_st_* = 0.401), have *F_st_* values > 2.5 SD above the mean. All other SNPs have *F_st_* values no more than 2 SD above the mean. Among those five SNPs with high *F_st_*, rs3811801 is located where a regulatory region of *ADH1B* might exist, and rs1042026 is in the intergenic region between *ADH1A* and *ADH1B*. The other three are all within the *ADH1B* locus. Positively selected alleles tend to accumulate in the top tail of the *F_st_* distribution [42], and genetic hitchhiking will also increase the frequencies of closely linked variants that might extend over long physical distance of low recombination [43]. Empirical studies also provide evidence showing that high *F_st_* could be driven by positive selection [44,45,46,47]. The human *ADH1B* locus, enriched with high *F_st_* variants, appears to be the site of natural selection favoring the *48His* allele at rs1229984, confirming previous conclusions with respect to the elevated frequency in East Asian populations.

Our previous studies concluded that extreme drift could not explain the allele frequency and *F_st_* as high as observed without invoking selection. Analyses of independent data by Yi Peng et al. [15] confirmed selection in East Asian populations using EHH and REHH. While a high *F_st_* value alone is not sufficient to justify the existence of local positive selection, independent studies concluding that the *ADH1B* Arg48His polymorphism has been the subject of selection in East Asia [15] indicate that local selection is a more likely explanation for the observed high *F_st_* values.

A different approach, the LRH analysis, has been applied to help assess our hypothesis about local selection on the *ADH1B* locus in Southwest Asian populations. The high EHH level over a large physical distance extending over 100 kb in both directions from the core haplotype TCGAGGC corresponds to a continuous increase of REHH level from the core, rendering a typical V shape in the REHH curve (Figure 5a). This is strong evidence showing that in Southwest Asia the haplotypes with the core TCGAGGC have low diversity, and the alleles on those haplotypes ride with the selection on the core to high frequencies. The simulated reference points give an overview of the strength of selection that an individual population has experienced (Figure 5b). ETJ, DRU, and YMJ show highest REHH values (> 99th percentile) both upstream (telomeric) and downstream (centromeric) of the core. ASH has REHH over 99th percentile upstream and over 95th percentile downstream of the core. Only SAM shows a REHH below the 95th percentile downstream of the core but above 95th percentile upstream.

The finding that Ashkenazi Jews share a more similar genetic background at the *ADH1B* locus to Southwest Asian populations than to European populations challenges the common belief that after so many years of admixture they are genetically closer to Europeans than to Southwest Asians despite their cultural ancestry. It is true that many other previously examined loci are indistinguishable in their SNP or haplotype profiles between Ashkenazi Jews and European populations, however, the *ADH1B* core haplotype TCGAGGC, which is common in Ashkenazi Jews and other Southwest Asian populations, is rare in populations with European origin. It seems that selection has somewhat preserved the original genetic profile on *ADH1B* locus in Ashkenazi Jews. Therefore, results from genetic analyses performed on Mixed Europeans (e.g., whites, Caucasians) should be applied to Ashkenazi Jews with caution, especially results from genes that might have been influenced by strong evolutionary forces before the migration of the Ashkenazi to Europe.

The core haplotype at *ADH1B* is 97 kb from the presumed telomeric hot-spot of recombination at *ADH7* [48]. That may be relevant to the decline seen in EHH; however, the decline in REHH is farther away at about 150 kb, suggesting that this presumed hot-spot may be more of a lukewarm site not relevant to the REHH during the time-period of the strong selection demonstrated.

Our data and analyses show for the first time strong evidence of selection on the 48His allele at *ADH1B*. The replication of the clear REHH V pattern in the three new population samples (ASH2, PAL, and ETJ2) provides strong support for our conclusion that selection operated on populations in Southwestern and Eastern Asian regions separately and independently, as the components of core haplotype extension are quite different. The remaining questions relate to the timing and strength of the selection as well as the nature of the selection. The enzyme defect caused by the 48His allele is clearly associated with resistance to alcoholism in modern populations [3,9,46,49] but that seems unlikely to be a selective factor in itself. The timing is poorly estimated but may roughly correspond to the Neolithic in Southwest Asia. The possible parallel with selection in East Asia offers a possible cause: agriculture or at least agriculture of a specific type. We showed previously that in East Asia the frequency of the 48His allele was higher in those ethnic groups that adopted agriculture earlier. Peng et al. [15] argued that rice cultivation was associated with the selection on the 48His allele. While there is general agreement that agriculture in East Asia is involved, the specific nature of the selective force is unknown. The evidence presented here strongly supports selection also having operated in Southwest Asia, but the specific nature of that selective force is similarly unknown. 

## Figures and Tables

**Table 1:** The two STRPs and the core haplotype SNPs (#60–66 in Figure 2) analyzed at *ADH1B* with their nucleotide positions, and ancestral/derived alleles (forward strand).

Position in Figure 2	Chromosome 4 SNPs, STRPs, Indel near *ADH1B*	Build 38 Nucleotide Position (Start-End for STRP, Indel)		SNP Alleles: Ancestral, Derived
		Start	End	
#54	rs12507573	99305167		A, C
	centromeric (TA)n	99305562	99305609	
#55	rs1042026	99307309		T, C
#60	rs1229984	99318162		C, T
#61	rs1159918	99321852		A, C
#62	rs6810842	99322288		G, T
#63	rs3811802	99323064		A, G
#64	rs3811801	99323162		G, A
#65	rs1693439	99324332		G, A
#66	rs9307239	99325780		C, T
#67	rs1789891	99329262		C, A
	telomeric (GTAT)n	99331657	99331709	
#68	rs36207960 dws 21 bp indel	99332232	99332252	

## Supplementary Materials

The following are available online at http://www.mdpi.com/2073-4425/9/9/452/s1, Table S1: The population samples—description, sample size, abbreviation, ALFRED UID (unique identifier) for population sample. Table S2: STRP haplotypes involving H7 and H5 are tabulated.
