# Supplementary material for: Recent Selection on a Class I ADH Locus Distinguishes Southwest Asian Populations Including Ashkenazi Jews
Source: Genes (Basel). 2018 Sep 7;9(9):452. doi: 10.3390/genes9090452 (PMC6162407; doi:10.3390/genes9090452)
Supplement: Supplementary file 1 [file genes-09-00452-s001.pdf]

Supplementary Table S1. **The population samples — description, sample size, abbreviation, ALFRED UID (unique identifier) for population sample.**

Sample Size N	Abbrev.	ALFRED population sample UID	Population description and Geographical region
			**Africa, Sub-Sahara**
67	BIA	SA000005F	Biaka
39	MBU	SA000006F	Mbuti
77	YOR	SA000036J	Yoruba, Nigeria
48	IBO	SA000099S	Ibo, Nigeria
38	HSA	SA000100B	Hausa, Nigeria
45	CGA	SA000487T	Chagga, Tanzania
20	MAS	SA000854R	Masai, Tanzania
88	AAM	SA000101C	African Americans
34	ETJ	SA000015G	Ethiopian Jews
21	ETJ2		Ethiopian Jews—independent sample
			**Southwest Asia, Europe**
41	YMJ	SA000016H	Yemenite Jews
101	DRU	SA000047L	Druze, Israel
40	SAM	SA000098R	Samaritans, Israel
70	PAL	SA002766V	Palestinian Arabs
109	ASH	SA000490N	Ashkenazi Jews
100	ASH2		Ashkenazi Jews—independent sample
53	ADY	SA000017I	Adygei
42	CHV	SA000491O	Chuvash
33	RUA	SA001530J	Russians, Archangelsk
46	RUV	SA000019K	Russians, Vologda
87	EAM	SA000020C	European Americans
109	IRI	SA000057M	Irish
51	DAN	SA000007H	Danes
34	FIN	SA000018J	Finns
			**Siberia**
47	KMZ	SA000489V	Komi Zyriane
50	KTY	SA000488U	Khanty
51	YAK	SA000011C	Yakut
			**East Asia**

54	KOR	SA000936S	Koreans
47	JPN	SA000010B	Japanese
55	CHS	SA000009J	Chinese, San Francisco, USA
50	CHT	SA000001B	Chinese, Taiwan
41	HKA	SA000003D	Hakka
24	CBD	SA000022E	Cambodians
40	AMI	SA000002C	Ami, Taiwan
42	ATL	SA000021D	Atayal, Taiwan
			**Pacific**
34	MCR	SA000063J	Micronesians
22	NAS	SA000012D	Nasioi, Solomon Islands
			**Americas**
54	NPA	SA000023F	Plains AmerIndians
50	SWA	SA000025H	Southwest AmerIndians
53	PMM	SA000026I	Pima, northern Mexico
48	MAY	SA000013E	Maya, Yucatan, Mexico
22	QUE	SA000069P	Quechua, Peru
65	TIC	SA000027J	Ticuna, Amazon region, Brazil
42	SUR	SA000014F	Rondonian Surui, Amazon region, Brazil
53	KAR	SA000028K	Karitiana, Amazon region, Brazil

Supplementary Table S2. STRP haplotypes involving H7 and H5 are tabulated. Population abbreviations are underlined where the estimated frequency is 9% or more for the haplotype. The four most frequently seen STRP haplotypes account for 87% of the STRP haplotypes containining the H5, H7 core SNP haplotypes.

STRP Haplotypes	Number	Present in these Populations by Regions		
		SW Asia	East Asia	Other
(TA)$_{17}$—H7—(GTAT)$_{11}$	287	ASH	YAK,CBD,CHS, CHT,HKA,KOR, JPN,AMI,ATL	ADY,CHV,RUV, MCR
(TA)$_{18}$—H7—(GTAT)$_{11}$	12	ASH	YAK,CHS,CHT, JPN,AMI,ATL	AAM,CHV
(TA)$_{17}$—H7—(GTAT)$_{12}$	5		CHS,HKA,JPN	
(TA)$_{16}$—H7—(GTAT)$_{11}$	2		KOR	
(TA)$_{26}$—H7—(GTAT)$_{11}$	1		CBD	
(TA)$_{19}$—H7—(GTAT)$_{11}$	1		KOR	
(TA)$_{17}$—H7—(GTAT)$_{13}$	1		JPN	
(TA)$_{17}$—H7—(GTAT)$_{14}$	1		CHS	
**H7 total=**	310			
(TA)$_{18}$—H5—(GTAT)$_{12}$	92	YMJ,DRU,ASH	YAK,CBD,CHS, CHT,HKA,JPN, AMI,ATL	EAM,MCR
(TA)$_{19}$—H5—(GTAT)$_{12}$	76	ETJ,YMJ,DRU, ASH	AMI,ATL	ADY,EAM
(TA)$_{20}$—H5—(GTAT)$_{12}$	25	ETJ,YMJ,DRU, SAM,ASH	HKA,ATL	RUA,MAY
(TA)$_{17}$—H5—(GTAT)$_{12}$	10	ETJ,ASH	CHS,CHT,KOR, JPN,AMI,ATL	
(TA)$_{19}$—H5—(GTAT)$_{13}$	10	DRU,SAM,ASH		
(TA)$_{18}$—H5—(GTAT)$_{13}$	8		AMI	
(TA)$_{21}$—H5—(GTAT)$_{12}$	7	ETJ,YMJ,DRU, ASH	CBD	
(TA)$_{26}$—H5—(GTAT)$_{12}$	4	YMJ,DRU,ASH	ATL	
(TA)$_{13}$—H5—(GTAT)$_{12}$	3	DRU,ASH		MAY
(TA)$_{14}$—H5—(GTAT)$_{12}$	2	DRU		EAM
(TA)$_{11}$—H5—(GTAT)$_{12}$	1			MAY
(TA)$_{17}$—H5—(GTAT)$_{13}$	1		CHS	
(TA)$_{18}$—H5—(GTAT)$_{14}$	1		HKA	
(TA)$_{19}$—H5—(GTAT)$_{11}$	1	ASH		
(TA)$_{20}$—H5—(GTAT)$_{11}$	1			EAM
(TA)$_{23}$—H5—(GTAT)$_{13}$	1	YMJ		
(TA)$_{25}$—H5—(GTAT)$_{11}$	1	ASH		
**H5 total=**	244			
